# ASPP2 inhibits tumor growth by repressing the mevalonate pathway in hepatocellular carcinoma

**DOI:** 10.1038/s41419-019-2054-7

**Published:** 2019-11-04

**Authors:** Beibei Liang, Rui Chen, Shaohua Song, Hao Wang, Guowei Sun, Hao Yang, Wei Jing, Xuyu Zhou, Zhiren Fu, Gang Huang, Jian Zhao

**Affiliations:** 1 Shanghai Key Laboratory of Molecular Imaging, Shanghai University of Medicine and Health Sciences, 201318 Shanghai, China; 2 Angecon Biotechnology Limited, 201318 Shanghai, China; 3 Transplantation Center, Changzheng Hospital, Second Military Medical University, 200011 Shanghai, China; 4 The Air Force Hospital from Northern Theater of PLA, Shenyang, 110041 China; 5 The Arctic Temple Clinic, Beijing Fourth Service Center, 8 Garden East Road, 100191 Beijing, China; 6 Changhai Hospital, Second Military Medical University, 200438 Shanghai, China

**Keywords:** Cancer metabolism, Gastrointestinal cancer

## Abstract

Cancer is, fundamentally, a disorder of cell growth and proliferation, which requires adequate supplies of energy and nutrients. In this study, we report that the haplo-insufficient tumor suppressor ASPP2, a p53 activator, negatively regulates the mevalonate pathway to mediate its inhibitory effect on tumor growth in hepatocellular carcinoma (HCC). Gene expression profile analysis revealed that the expression of key enzymes in the mevalonate pathway were increased when ASPP2 was downregulated. HCC cells gained higher cholesterol levels and enhanced tumor-initiating capability in response to the depletion of ASPP2. Simvastatin, a mevalonate pathway inhibitor, efficiently abrogated ASPP2 depletion-induced anchorage-independent cell proliferation, resistance to chemotherapy drugs in vitro, and tumor growth in xenografted nude mice. Mechanistically, ASPP2 interacts with SREBP-2 in the nucleus and restricts the transcriptional activity of SREBP-2 on its target genes, which include key enzymes involved in the mevalonate pathway. Moreover, clinical data revealed better prognosis in patients with high levels of ASPP2 and low levels of the mevalonate pathway enzyme HMGCR. Our findings provide functional and mechanistic insights into the critical role of ASPP2 in the regulation of the mevalonate pathway and the importance of this pathway in tumor initiation and tumor growth, which may provide a new therapeutic opportunity for HCC.

## Introduction

Dysregulation of a wide range of metabolic pathways can be involved in carcinogenesis^1^. For example, cholesterol has a critical role in maintaining the stability and architecture of the plasma membrane and is needed by highly proliferative cancer cells^2^. Tumor cell membranes have been found to be enriched in cholesterol, suggesting that enhanced cholesterol utilization is an important feature of malignant and perhaps metastatic tumors^3^. Mutational data from The Cancer Genome Atlas (TCGA) project also implicates cholesterol metabolism in cancer development^4^. The mevalonate pathway (MVA) is the essential metabolic pathway that converts acetyl-CoA to sterols such as cholesterol and non-sterol isoprenoids. Clinical and experimental findings suggest that malignant cells and tissues need higher amounts of cholesterol and intermediates of the mevalonate pathway than their normal counterparts^5,6^. Some types of cancers, such as hepatocellular carcinoma (HCC), depend on mevalonate pathway for growth. For example, inhibition of mevalonate metabolism suppresses tumor initiation and growth in a *myc*-transgenic murine HCC model^7^, activation of the mevalonate pathway also plays an essential role in liver tumorigenesis caused by p53 loss in mice^8^.

The mevalonate pathway is tightly regulated by a series of enzymes. 3-hydroxy-3-methylglutaryl-CoA reductase (HMGCR) is the rate-limiting enzyme, and can be blocked by statins^9^; indeed, such inhibition has antitumor effects in multiple tumor types^9^. Clinical observations have shown a negative association between the use of statins and the risk of developing liver cancer^10^.

Transcriptional activation of key rate limiting enzymes, like HMGCR, in mevalonate pathway is controlled by sterol-regulated cleavage of the membrane-bound transcription factor SREBP-2 (sterol regulated element binding protein-2)^11–13^. Depletion of intracellular sterols enhances SREBP-2 translocation from the endoplasmic reticulum (ER) to the Golgi apparatus, leading to subsequent cleavage to its active mature form and nuclear translocation^14^. SREBP-2 binds to sterol regulatory elements (SREs) in its target gene promoters to activate their transcription.

ASPP2 is a member of the ankyrin-repeat, SH3-domain, and proline-rich region-containing protein (ASPP) family, and is a haplo-insufficient tumor suppressor^15,16^. Aberrant expression of ASPP2 has been found in various human cancers^17^. Our previous study found that ASPP2 is downregulated by DNA methylation in HCC^18^. ASPP2 inhibits tumor growth and metastasis through regulation of apoptosis, autophagy, and epithelial plasticity^16,19–21^. However, it remains unknown whether ASPP2 is involved in the regulation of mevalonate metabolism, which may contribute to its inhibitory effect on tumor progression. In this study, we provide evidence that downregulation of ASPP2 enhances tumor-initiating capabilities and tumor growth by promoting mevalonate metabolism in HCC.

## Results

### Downregulation of ASPP2 enhances mevalonate pathway gene expression and cholesterol biosynthesis in HCC cells

**Fig. 1:**
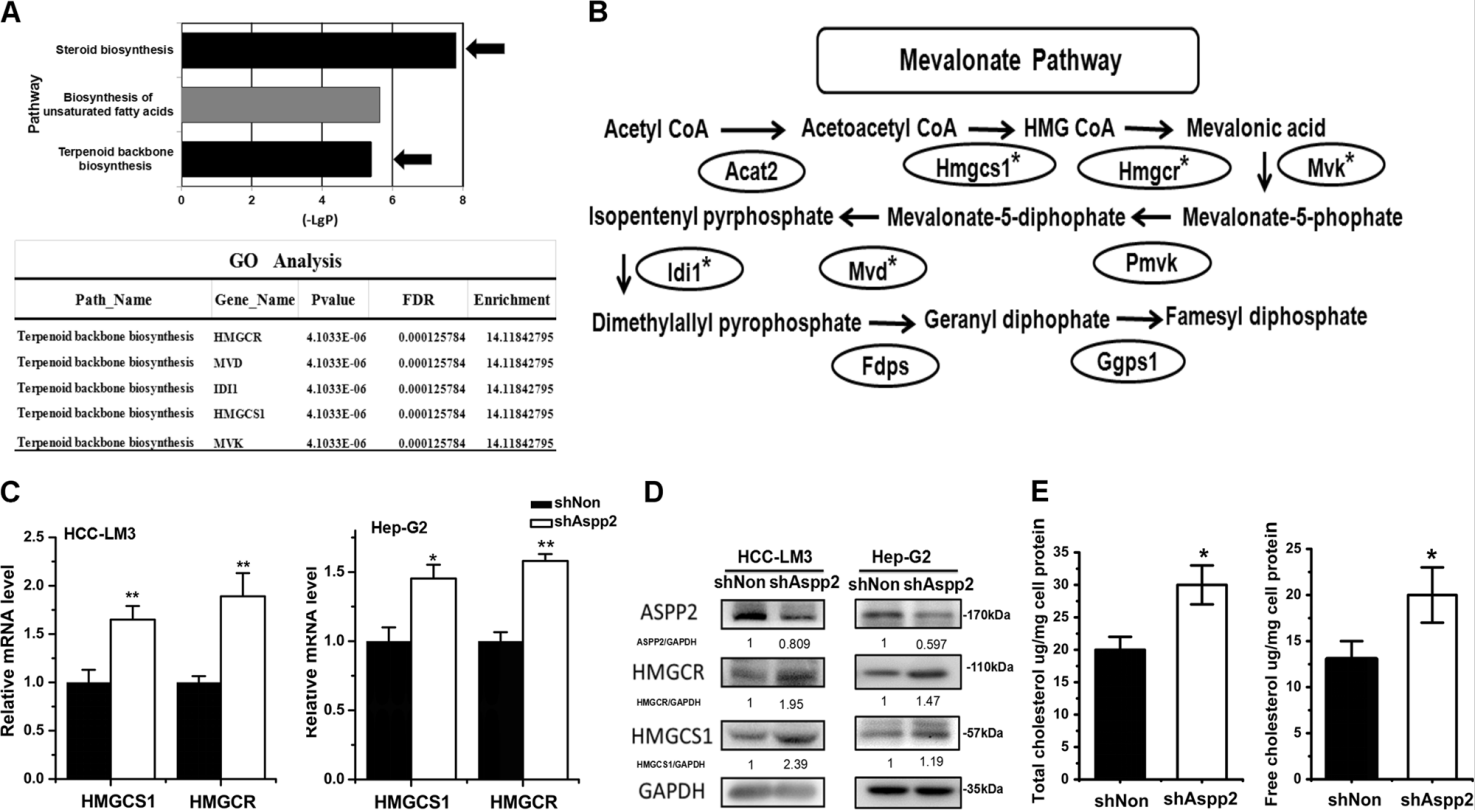
Downregulation of ASPP2 enhances mevalonate pathway gene expression and cholesterol biosynthesis in HCC cells. **a** Top: KEGG Pathway analysis of ASPP2 knockdown show significant differential changes (*P* < 0.05) in canonical metabolic pathways. The *P* value for each pathway is indicated by the bar and is expressed as −1 times the log of the *P* value. Arrows are used to indicated the two pathways closely related with mevalonate metabolism. Bottom: Gene Ontology (GO) analysis revealed genes were most significantly upregulated and enriched by ASPP2 knockdown in the terpenoid backbone biosynthesis pathway (*P* < 0.05, FDR < 0.25). **b** Schematic of the mevalonate pathway with asterisk (*) marking upregulated genes by ASPP2 knockdown in panel (**a**) bottom. **c** qRT-PCR of Hmgcs1, Hmgcr mRNA expression in HCC-LM3 and HepG2 cells infected with lentivirus encoding shRNA was indicated for 72 h. β-actin was used as a control. **d** Corresponding western-blot of HMGCS1 and HMGCR in HCC-LM3 and HepG2 cells. **e** Total cholesterol and free cholesterol were measured by GC-MS methods, using the internal standard strategy as methods described. Each symbol represents the mean ± SD of three wells. Asterisk (*) indicates *P* < 0.05

To explore the mechanisms driving the inhibitory effects of ASPP2 on tumor growth and progression, we performed a microarray analysis to identify the potential down-stream targets of ASPP2 by comparing gene expression in ASPP2-depleted HCC-LM3 cells and parent cells. Interestingly, GO analysis revealed a significant negative correlation between ASPP2 expression and terpenoid backbone biosynthesis and steroid biosynthesis (Fig. 1a). Expression of genes involved in mevalonate pathway, such as Hmgcs1, Hmgcr, Mvk, Mvd, and Idi1, appeared to be significantly enriched (*P* < 0.05, FDR < 0.25) in ASPP2-depleted HCC-LM3 cells (Fig. 1b). To validate this finding, we examined the expression of HMG-CoA reductase (HMGCR) and HMG-CoA synthase1 (HMGCS1), which are the rate-limiting enzymes for mevalonate pathway^22,23^, by real-time PCR and Western Blotting. The results revealed that the mRNA and protein levels of HMGCR and HMGCS1 were notably elevated in ASPP2-depleted HCC-LM3 and HepG2 cells (Figs. 1c and d). Moreover, knockdown of ASPP2 led to a higher levels of free cholesterol and total cholesterol in HCC-LM3 cells by GC-MS analysis (Fig. 1e). Taken together, these observations suggest that downregulation of ASPP2 in HCC cells enhances mevalonate pathway and cholesterol biosynthesis.

### Mevalonate metabolism is essential for maintaining tumor-initiating capability in ASPP2-depleted HCC cells

**Fig. 2:**
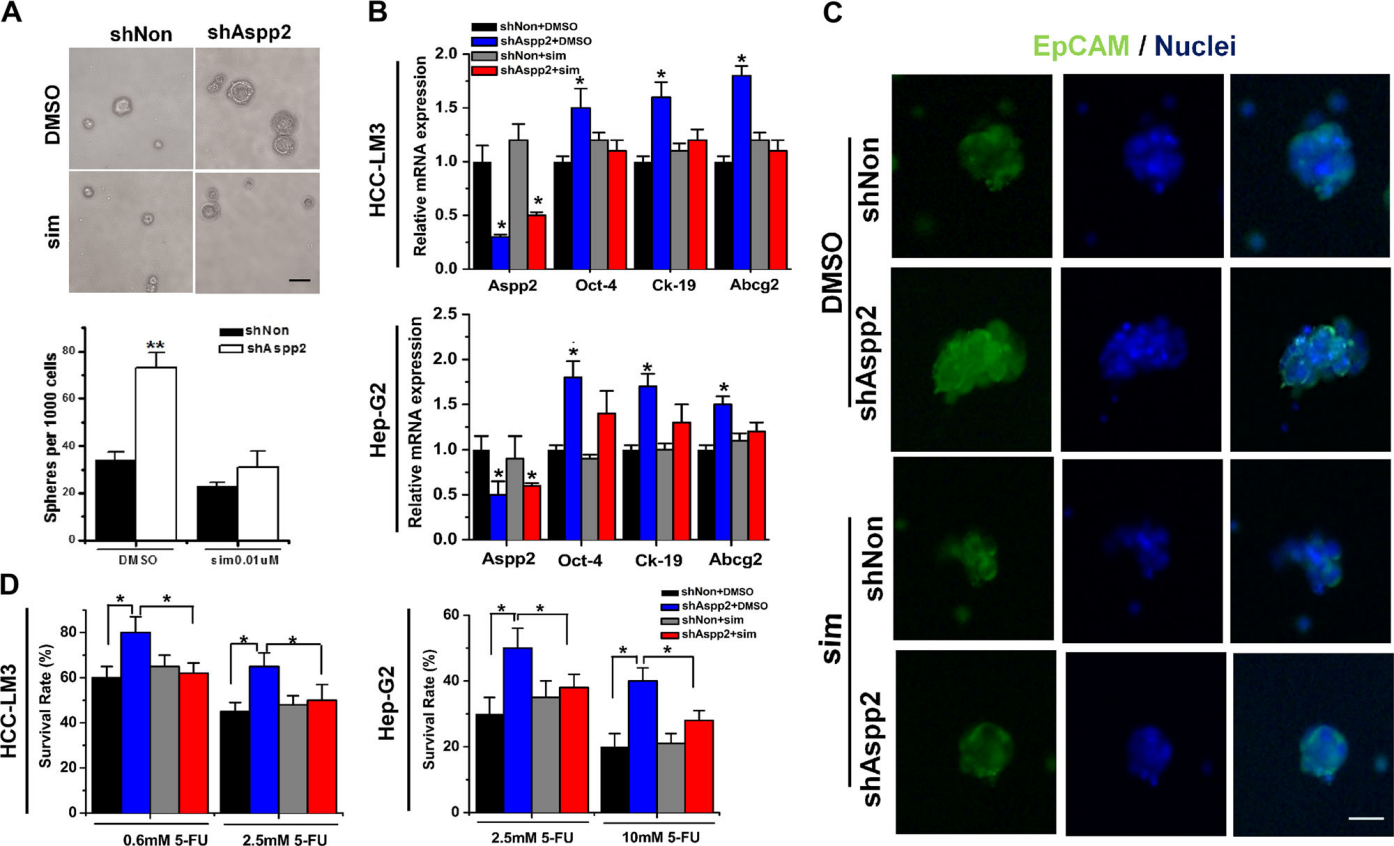
Mevalonate metabolism is essential for maintaining tumor-initiating capability in ASPP2-depleted HCC cells. **a** Top: Representative images of spheroid formation in HCC-LM3 cells treated with 0.01 μM simvastatin or DMSO after knock down of ASPP2. Bottom: the number of spheres formation per 1000 HCC-LM3 cells.Scale bar, 100 mm. **b** qRT-PCR gene expression of Aspp2, Oct-4, Ck-19, and Abcg2 in HCC-LM3 and HepG2 cells transfected with the indicated lentivirus and treated with 10 μM simvastatin or DMSO. **c** Immunofluorescence stainings of HCC-LM3 spheres formed from cells transfected with the indicated lentivirus and treated with 10 μM simvastatin or DMSO. Green staining represented EpCAM protein. DAPI was used stain nuclear(blue staining) Scale bar, 40 mm **d** Cell viability of the infected HCC-LM3 and HepG2 cell treated with 10 μM simvastatin or DMSO and 5-FU(0.6 mM,2.5 mM or 10 mM) was analyzed by MTS assay. Asterisk (*) indicates *P* < 0.05, asterisks (**) indicates *P* < 0.01

Mevalonate metabolism disorder has been linked to the development and tumor-initiating capability in various cancers^24–26^. We have previously demonstrated that downregulation of ASPP2 conferred HCC cells with stem cell-like properties^27^. To verify the importance of mevalonate metabolism in ASPP2-depletion induced tumor-initiating capability in HCC cells, we inhibited mevalonate metabolism with simvastatin, a type of statin that can inhibit HMGCR to deplete precursors of the mevalonate pathway and lower cholesterol levels^28,29^. Treatment with simvastatin had no effect on cell proliferation in HCC-LM3 grown in monolayer culture (Supplementary Fig. 1). However, in a suspension culture system, the numbers and the size of cell spheres were greatly decreased in simvastatin-treated ASPP2-depleted HCC-LM3 cells (Fig. 2a), suggesting decreased self-renewal. EpCAM is considered as a putative maker for tumor-initiating cells in HCC^30^, as EpCAM-positive HCC cells possess progenitor cell features^31^. Simvastatin supplementation abrogated ASPP2 depletion-induced EpCAM mRNA and protein expression in HCC-LM3 cells (Supplementary Fig. 2), as well as EpCAM expression in the surface of HCC-LM3 spheres (Fig. 2c). In addition, we assessed the expression of stemness-associated genes by qRT-PCR. ASPP2 depletion resulted in upregulation of transcription factor Oct4, ATP-binding cassette transporter Abcg2, and Ck-19, a putative marker of tumor-initiating HCC cells, in HCC-LM3 and HepG2 cells. These alterations were compromised by simvastatin treatment (Fig. 2b). Resistance to chemotherapy has been regarded as another property of tumor initiating cells. Compared to control cells, ASPP2-depleted HCC-LM3 and HepG2 cells displayed significantly higher resistance to 5-FU treatment. However, this resistance was decreased by simvastatin (Fig. 2d).

**Fig. 3:**
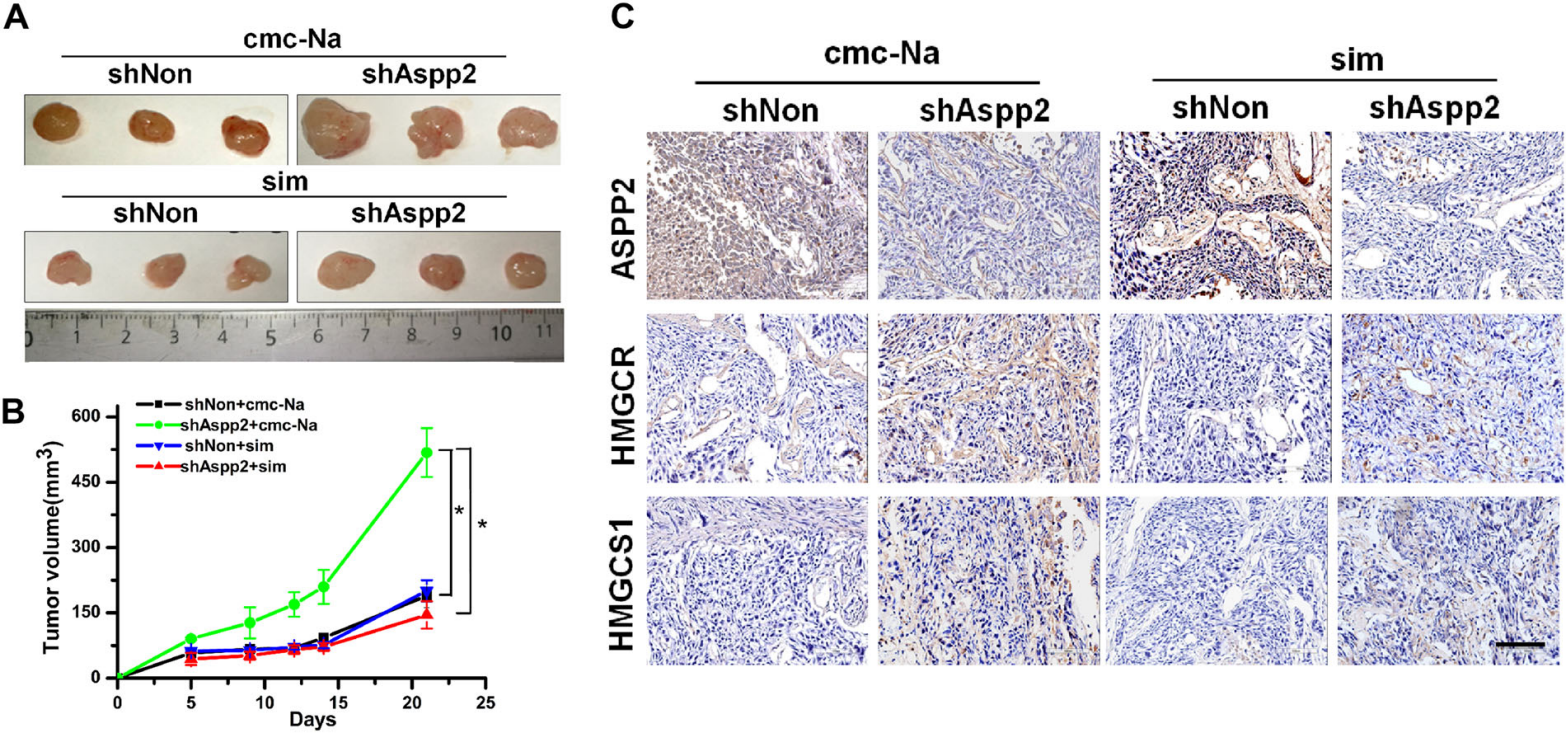
Downregulation of ASPP2 promoted tumor growth in vivo by activating mevalonate biosynthesis. **a** Representative dissected tumors from nude mice treated without (top) or with (bottom) simvastatin for 3 weeks and corresponding volume measurement, **b** Asterisk (*) indicates *P* < 0.05. **c** Immunohistochemical staining of tumors derived from nude mice for ASPP2, HMGCR, and HMGCS1 (magnification, ×200). ASPP2-silenced groups exhibited increased HMGCR and HMGCS-1 expression. These alterations were compromised in ASPP2-silenced groups treated with simvastatin

To further confirm that the mevalonate pathway contributes to tumor growth of ASPP2-depletion HCC cells in vivo, HCC-LM3 cells expressing shASPP2 or shNon were injected into the flank of nude mice, treated with or without simvastatin. Twenty one days after the xenograft, simvastatin significantly delayed tumor growth in ASPP2-silenced groups (Fig. 3a, b). In addition, immunohistochemical staining showed tumors originated from ASPP2-silenced groups exhibited increased HMGCR and HMGCS-1 expression (Fig. 3c). These data demonstrated that the mevalonate pathway is critical for maintaining tumor-initiating capability in ASPP2-depleted HCC cells.

### ASPP2 interacts with SREBP-2 to regulate mevalonate metabolism

**Fig. 4:**
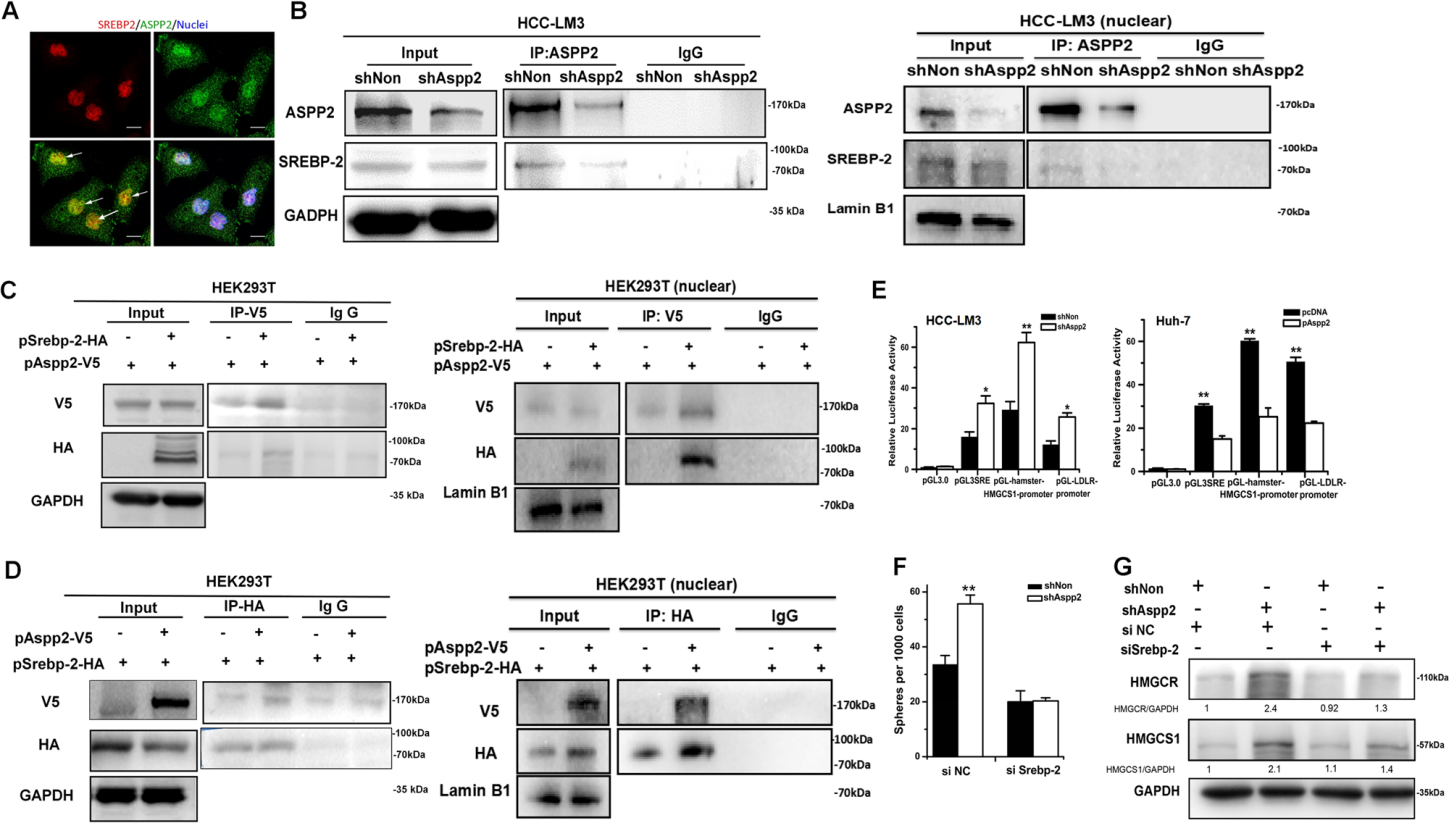
Downregulation of ASPP2 promotes mevalonate metabolism by activation of SREBP-2. **a** Immunofluorescent images of HCC-LM3 cells showing the cellular location of endogenous ASPP2 and SREBP-2. ASPP2 and SREPB-2 were stained green and red, respectively. DAPI (blue stain) was used to stain the nucleas.Scale bars: 40 μm. **b** Western blot of the immunoprecipitated ASPP2-SREBP-2 complex in total cell lysates (Left) or nuclear proteins lysates (Right) with anti-ASPP2 in HCC-LM3 cells. **c** Western blots of V5-tagged ASPP2 co-transfected with HA-SREBP-2 into HEK293T following IP with anti-V5 antibody in total cell lysates (Left) and nuclear proteins lysates (Right). **d** Western blots of HA-tagged SREBP-2 co-transfected with ASPP2-V5 into HEK293T following IP with anti-HA antibody in total cell lysates (Left) and nuclear proteins lysates (Right). **e** Luciferase activity of Hamster HMGCS1, human LDLR promoters and three Sterol regulatory elements (3SRE) with respect to reporter vector pGL3.0. The constructs were co-transfected with an internal control pRL-TK vector into HCC-LM3 cells with silencing of ASPP2 (left) and Huh-7 cell with ASPP2 overexpression (right). **f** The number of spheres per 1000 HCC cells with siRNA negative control or siRNA Srebp-2 after knock down of ASPP2. **g** Western blot of HMGCR and HMGCS1 protein in HCC-LM3 cells infected with LV-shASPP2 or LV-shNon and treated with siAspp2 and/or siSrebp2. Asterisk (*) indicates *P* < 0.05, asterisks (**) indicates *P* < 0.01

Microarray analysis revealed that several mevalonate pathway genes appeared to be transcriptionally upregulated in ASPP2 depleted HCC-LM3 cells (Fig. 1a), and we hypothesized that ASPP2 is an upstream regulator of mevalonate pathway, probably through regulation of SREBP-2. It has been demonstrated that SREBP-2 is a predominant regulator of the mevalonate pathway^32^. SREBP-2 is located entirely in the nucleus of HCC-LM3 cells, and ASPP2 could be detected in both the nucleus and cytoplasm in HCC-LM3 cells (Fig. 4a). Interestingly, we found SREBP-2 co-localized with endogenous ASPP2 in the nucleus of HCC-LM3 cells (Fig. 4a). Moreover, endogenous ASPP2 was found to interact with SREBP-2 in HCC-LM3 as detected by co-immunoprecipitation in total cell lysates or nuclear lysates (Fig. 4b). The separation of nuclear proteins was confirmed by the low expression of cytoplasmic protein α-tubulin and the high expression of nuclear protein Lamin B1 (Supplementary Fig. 3). Depletion of ASPP2 did not reduce the protein level of SREBP-2, however the interaction between ASPP2 and SREBP-2 was greatly reduced (Fig. 4b). This interaction was further confirmed by ectopic expression of V5-tagged ASPP2 and HA-tagged SREBP-2 in HEK293T cells (Fig. 4c, d). To explore whether ASPP2 regulates SREBP-2 transcriptional activity, we generated luciferase reporter constructs containing the hamster HMGCS1 promoter, the human LDLR promoter, or three sterol regulatory elements (3SRE) that bind SREBP-2^13^. Ectopic expression of ASPP2 in HCC-LM3 and Huh-7 greatly reduced 3SRE, HMGCS1 and LDLR promoter activities, while depletion of ASPP2 had the reverse effect on their activities (Fig. 4d). ASPP2 depletion-induced HMGCR and HMGCS-1 expression were ablated when *Srebp-2* was knocked down (Fig. 4f). Moreover, siRNA targeting *Srebp-2* abrogated ASPP2 depletion-induced sphere formation in HCC-LM3 cells (Fig. 4e). These results suggested that ASPP2 interacts with SREBP-2 and serves as a negative regulator of mevalonate metabolism.

### ASPP2-regulated mevalonate metabolism is associated with tumor progression and provides prognostic value

**Fig. 5:**
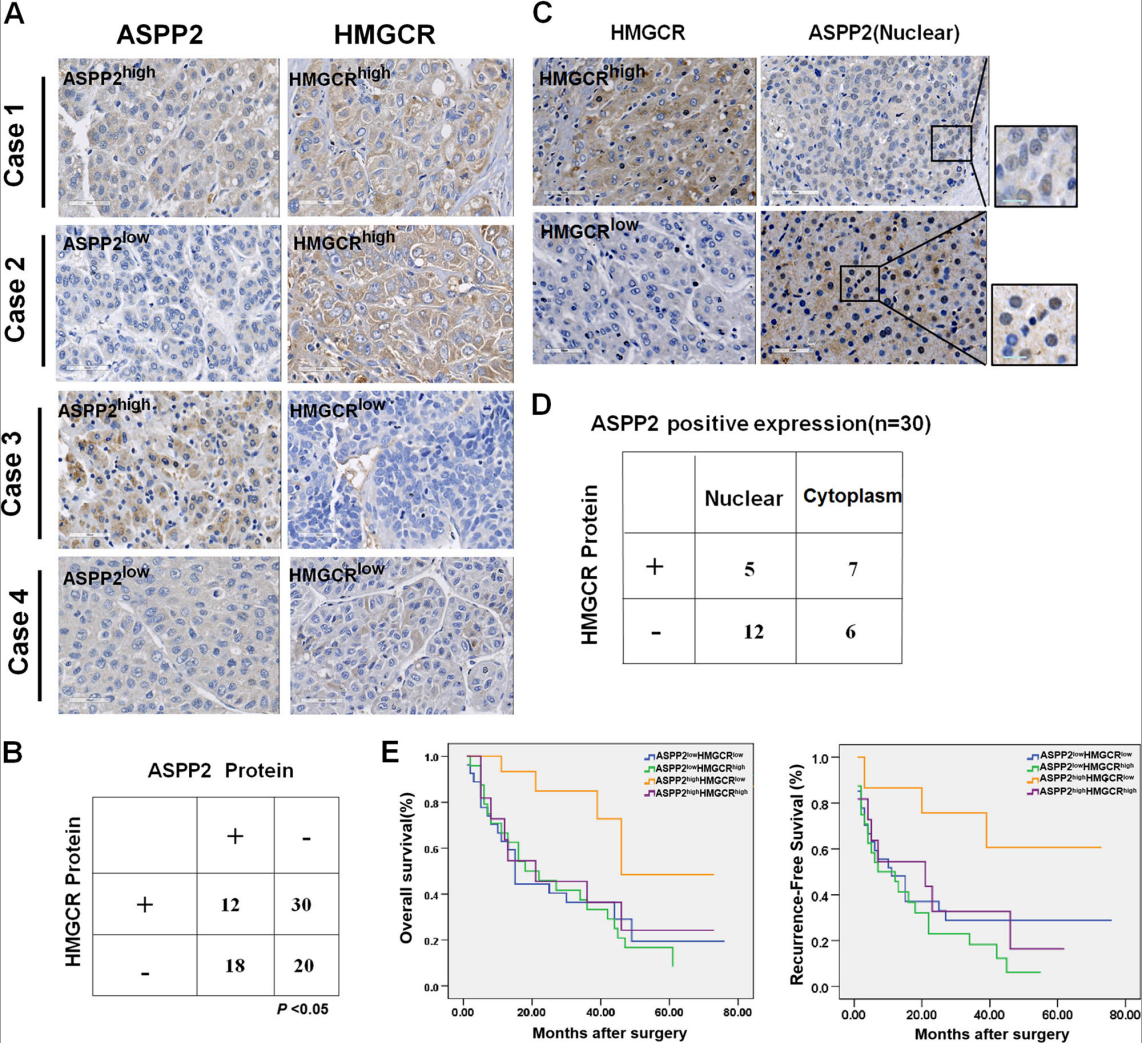
Expression of ASPP2 correlates negatively with HMGCR in surgical specimens of HCC. **a** ASPP2 and HMGCR were detected by immunohistochemical staining in 80 HCC samples. Representative pictures are shown for four patients. (Scale bars: 50 μm). **b** Table of ASPP2 and HMGCR high and low expression according to immunohistochemistry scoring. Significant negative correlation between ASPP2 and HMGCR expression was found, Fisher’s test *P* < 0.05. **c** The strong expression of ASPP2 in nuclear in 17 HCC samples. Representative pictures of immunohistochemical staining are shown for two patients.Scale bars: 50 μm. **d**. Table of positive ASPP2 expression in nuclear or cytoplasm and their HMGCR expression according to the scores of immunohistochemistry staining. **e** Left: Overall survival (OS) and right: recurrence-free survival (RFS) rate curves of the analyzed four subgroups (ASPP2 low/HMGCR low; ASPP2 low/HMGCR high; ASPP2 high /HMGCR low; ASPP2 high/HMGCR high), (*n* = 80). Kaplan-Meier analysis showed RFS (*P* < 0.05) and OS (*P* < 0.05) were significantly best among patients with ASPP2-high and HMGCR-low expression

To assess the clinical impact of ASPP2-regulated mevalonate metabolism, we examined ASPP2 and HMGCR protein expression in 80 HCC tissues by immunohistochemistry. The clinical pathological parameters of all HCC patients are shown in Supplementary Table 1. In 30 cases, the tumor samples displayed high ASPP2 expression. Most of the ASPP2 was located in the cytoplasm (Fig. 5a), but 17 cases had strong nuclear staining of ASPP2 (Fig. 5c). Thirty-eight percent (30/80) cases showed low ASPP2 expression and high expression of HMGCR, while 23% (18/80) cases showed high ASPP2 expression and low HMGCR expression. Thus, the expression of ASPP2 and HMGCR is inversely correlated (*P* < 0.05; Fig. 5b). Among the cases with ASPP2 in the nucleus, 70.6% (12/17) had low HMGCR expression, in contrast to only 46.2% (6/13) of cases with cytoplasmic ASPP2 (Fig. 5d). This suggests that nuclear ASPP2 may be more likely to negatively regulate HMGCR expression.

The relationships between ASPP2 and HMGCR expression and clinical features are statistically analyzed in Table 1. A significant correlation between HMGCR expression and tumor volume was observed (*P* = 0.003): patients with high HMGCR expression were prone to have a larger tumor volume. Further, patients with ASPP2-high and HMGCR-low expression had smaller tumor volumes (*P* = 0.008) and less cirrhosis (*P* = 0.018). Consistent with these findings, patients with ASPP2-high and HMGCR-low expression exhibited the best recurrence-free survival (RFS), as well as overall survival (OS) (Fig. 5e). These data suggest that ASPP2-regulated mevalonate metabolism contributes to tumor progression in HCC.

**Table 1:** The associations of ASPP2 and HMGCR expression with clinicopathologic characteristics in 80 patients with HCC

	Whole study (*n* = 80)			ASPP2 negative group (*n* = 50)			ASPP2 positive group (*n* = 30)		
	HMGCR expression		*P*	HMGCR expression		*P*	HMGCR expression		*P*
	Negative (*n* = 38)	Positive (*n* = 42)		Negative (*n* = 20)	Positive (*n* = 30)		Negative (*n* = 18)	Positive (*n* = 12)	
Sex									
Male	30	29	0.315	14	19	0.625	16	10	1.0
Female	8	13		6	11		2	2	
Age (years)									
<50	11	21	0.055	4	17	0.023	7	4	1.0
≥50	27	21		16	13		11	8	
HBsAg									
Negative	6	8	0.702	3	7	0.718	3	1	0.632
Positive	32	34		17	23		15	11	
AFP (ng/ml)									
≤400	23	18	0.114	12	13	0.248	11	5	0.457
>400	15	24		8	17		7	7	
Tumor volume (cm^3^)									
≤5	28	17	0.003	14	14	0.103	14	3	0.008
>5	10	25		6	16		4	9	
Cirrhosis									
−	13	10	0.305	3	9	0.224	10	1	0.018
+	25	32		17	21		8	11	
AJCC stage									
Stage I–II	15	19	0.602	7	15	0.295	8	4	0.709
Stage III–IV	23	23		13	15		10	8	
Recurrence time (months)				85					
≤6	11	17	0.28	7	13	0.556	4	4	0.678
>6	27	25		13	17		14	8	

Note: *P* values are two-tailed and based on the Pearson *χ*^2^ test

In univariate analysis, tumor size, AJCC stage, ASPP2, and HMGCR expression status are to be prognostic factors for RFS and OS (*P* < 0.05, Table 2). In multivariate analysis, tumor size, ASPP2, and HMGCR expression status were shown as significant independent predictors of RFS and OS (Table 3). Patients with HMGCR-high expression were 2.530 times more likely to suffer from relapse than patients with HMGCR-low expression (hazard ratio, 2.530; 95% confidence interval, 1.355–4.725). Patients with ASPP2-high expression were about 0.453 times less at risk of relapse than patients with ASPP2-low expression (hazard ratio, 0.453; 95% confidence interval, 0.235–0.873). Therefore, increased expression of HMGCR with decreased ASPP2 may serve as a prognostic indicator for patients with HCC.

**Table 2:** Univariate analyses of factors associated with recurrence-free survival and overall survival

Variables	RFS		OS	
	Hazard ratio (95% CI)	*P*	Hazard ratio (95% CI)	*P*
Gender (male vs. female)	0.670 (0.351–1.278)	0.224	0.67 (0.348–1.275)	0.22
Age, years (≥50 vs. <50)	0.630 (0.364–1.089)	0.098	0.776 (0.481–1.203)	0.363
HbsAg (positive vs. negative)	1.181 (0.425–3.285)	0.75	1.078 (0.385–3.013)	0.887
AFP, ng/ml (>400 vs. ≤400)	1.713 (0.988–2.970)	0.055	1.519 (0.876–2.634)	0.136
Cirrhosis (yes vs. no)	1.589 (0.871–2.899)	0.131	1.436 (0.724–1.894)	0.239
Tumor size, cm (≥5 vs. <5)	3.124 (1.692–5.768)	0.00	2.79 (1.523–5.110)	0.001
AJCC stage (III–IV vs. I–II)	2.080 (1.108–3.903)	0.023	1.981 (1.056–3.717)	0.033
ASPP2 (high vs. low)	0.423 (0.221–0.809)	0.009	0.437 (0.229–0.833)	0.012
HMGCR (high vs. low)	2.095 (1.193–3.679)	0.01	1.979 (1.130–3.466)	0.017

**Table 3:** Multivariate analyses of factors associated with recurrence-free survival and overall survival

	Hazard ratio (95% CI)	*P*
RFS		
Tumor size, cm (≥5 vs. <5)	2.530 (1.355–4.725)	0.004
ASPP2 (high vs. low)	0.505 (0.257–0.989)	0.026
HMGCR (high vs. low)	2.182 (1.147–4.150)	0.017
OS		
Tumor size, cm (≥5 vs. <5)	2.103 (1.122–3.942)	0.02
ASPP2 (high vs. low)	0.453 (0.235–0.873)	0.017
HMGCR (high vs. low)	2.530 (1.355–4.725)	0.004

Multivariate analysis, cox proportional hazards regression model

Variables were adopted for their prognostic significance by univariate analysis and no obvious correlation between each other

## Discussion

Here we demonstrate that ASPP2, an activator of p53, regulates tumor-initiating capability and tumor growth by inhibiting SREBP-2-mediated mevalonate metabolism in HCC. HCC cells gained higher cholesterol levels, increased cancer stemness characters and tumor growth by lentivirus-mediated downregulation of ASPP2. Simvastatin, a mevalonate metabolism pathway inhibitor reversed the increased tumor growth and stemness characteristics of ASPP2-depleted cells.

p53, the gatekeeper for cell growth and division, acts via multiple pathways, such as cell-cycle arrest, cell death and senescence. Increasing evidence demonstrates that regulation of cellular metabolic pathways by p53 contributes to its tumor suppressor effects^33^. Notably, recent evidence shows that p53 represses mevalonate metabolism via inhibiting maturation of SREBP-2, which plays a crucial role in p53-mediated tumor suppression in a mouse model of liver tumorigenesis^8^. Moreover, p53 harboring cancer-derived missense mutations interacts with SREBP-2 to activate the mevalonate pathway, and this is implicated in maintaining a malignant phenotype in breast cancer^34^.

ASPP2 was first identified as an activator of the p53 family that regulates apoptosis^35^. Subsequently, ASPP2 was found to regulate a wide range of biological events by cooperating with specific partners, such as p73, Bcl-2, NF-kB, Yes-associated protein-1, RAS, Par3, β-catenin, and Beclin-1. At cell membranes, through its N-terminus ASPP2 binds Par3 to maintain the integrity of cell polarity^36,37^, and binds the β-catenin-E-cadherin complex to regulate epithelial plasticity^20^. In the cytoplasm, ASPP2 binds RAS to regulate RAS signaling^38,39^, and interacts with ATG5 and Beclin 1 to regulate autophagy^19,21^. In the nucleus, ASPP2 binds p53 and p73 to stimulate apoptosis by its C terminus^40^. Phosphorylation of ASPP2 by the RAS/Raf/MAPK pathway causes the translocation of ASPP2 from the cell membrane to cytosol/nucleus^41^. LPS stimulates ASPP2 translocation from the cytoplasm to nucleus in murine macrophages, and causes nuclear accumulation of ASPP2 at the blood–cerebral spinal fluid barrier in an LPS-induced maternal inflammation mouse model^42^. The C terminus of the ASPP2 contains ankyrin repeats, which may facilitate ASPP2 to enter the nucleus through the RanGDP/Ankyrin Repeats binding nuclear import pathway^43^.

Here, we showed evidence that ASPP2 interacts with SREBP-2 in the nucleus and inhibits the transcriptional activation of mevalonate pathway genes by SREBP-2. HCC tissues with nuclear ASPP2 staining were more prone to have low HMGCR expression than HCC tissue with cytoplasmic ASPP2 staining, further supporting a negative effect of nuclear ASPP2 on the mevalonate pathway.

Previous observations in cancer cells have shown ASPP2 expression predominantly in the cytoplasm or at the cell membrane^20^. However, our datas showed that ASPP2 expression was both nuclear and cytoplasm in HCC-LM3 cells and in about 21% (17/80) of cases of HCC. Co-localization of ASPP2 and SREBP-2 in HCC-LM3 nucleus was observed. Previous studies have shown that the transcriptional activity of SREBP-2 could be regulated by interaction with mutant p53, Myc, and RB^9^. Two gain-of-function p53 mutations are able to interact with nuclear SREBP-2 and increase SREBP-2-mediated transcription of the mevalonate pathway genes^34^. Data from the Encyclopedia of DNA Elements (ENCODE) project has shown that MYC binds to promoters of mevalonate pathway genes in close proximity to SREBP-2, which may contribute to Myc-induced hepatocarcinogenesis. RB prevents the association of SREBP-2 with target gene promoter and serves as a negative regulator of mevalonate metabolism^44^. Our finds suggest that ASPP2 is a novel negative regulator of SREBP-2. Given that ASPP2 interacts with p53 in the nucleus, the interaction between of ASPP2 and SREBP2 is p53 dependent or not still need further investigation.

Since the liver is responsible for about 80% of de novo cholesterol biosynthesis in humans, it is not surprising to see a critical role of the mevalonate pathway in the development of liver cancer. Our findings have identified a crucial role of ASPP2 in regulating mevalonate metabolism and its importance in tumor-initiating capability and tumor growth, which may provide novel therapeutic opportunities for HCC.

## Materials and methods

### Cell culture

We purchased HEK293T cells from American Type Culture Collection (ATCC). HCC-LM3 (p53 wt), HepG2 (p53 wt), and Huh-7 (p53 mut) were obtained from the Liver Cancer Institute, Zhong Shan Hospital, Fudan University (Shanghai, People’s Republic of China). All cells line had been identified and tested the mycoplasma contamination. These cells were maintained in DMEM (Gibco, Waltham, MA, USA) supplemented with 10% (vol/vol) FBS (Gibco) and kept at 37 °C and 5% CO2. Simvastatin was used to inhibit HMGCR to deplete precursors of the mevalonate pathway and lower cholesterol levels.

### Lentivirus shRNA and small interfering RNA construction

We designed three pairs of cDNA oligonucleotides targeting ASPP2 mRNA expression, using web-based software from Invitrogen and InvivoGen Inc. After synthesis, we inserted these double-strand oligos into the vector pENTR/U6 (Invitrogen) and sequenced the resulting plasmids to ensure the shRNA construct targeted human ASPP2 expression or were scrambled, which were generated and designated as LV-shASPP2 and LV-shNon. Then, the plasmids were transfected into HCC-LM3 cells and gene silencing efficiency was validated 48 h after transfection by real-time PCR and Western blot. Details about lentivirial vector production and lentivirus infection can be found in the supplementary material.

### Cholesterol analysis

HCC-LM3 cells in 6-well plates were infected with LV-shNon and LV-shAspp2. After three days, two group cells were collected. Free cholesterol and total cholesterol were measured by GC-MS methods, using the internal standard strategy. Each symbol represents the mean ± SD of three wells. The experiments have been repeated at least three times.

### Luciferase reporter plasmids construction and luciferase reporter assays

The luciferase reporter plasmids, pGL-hamster-HMGCS1-promoter and pGL-LDLR-promoter were designed and generated (details can be found in the supplementary material). The pGL3.0-enhancer (Promega) and the constructed luciferase reporter plasmids were transfected into HCC (3 × 10^4^) cells in 48-well plates with the pRL-TK in triplicate by X-tremeGENE HP DNA Transfection Reagent. Then the cells were collected after 24 h transfection to measure the luciferase activities with the Dual Luciferase Reporter Assay System (Promega). All data are presented as the means ± S.D. The experiments have been repeated at least three times.

### Tumor xenograft model

After infection with LV-shNon and LV-shAspp2 (at a MOI of 50), HCC-LM3 cells (5 × 10^6^) were implanted into the flank of nude mice by subcutaneous injection (male BALB/c nu/nu, six in each group). Tumors were well-established after 7 days. Simvastatin (40 mg/kg body weight) in 0.5% CMC-Na (sodium carboxymethyl cellulose) was given to mice daily by intraperitoneal injection for 3 weeks. Then the mice were sacrificed and the tumor tissues were isolated for histopathology experiments.

### Sphere formation assay

0.4% bovine serum albumin, 4 μg/mL insulin (Sigma-Aldrich, St Louis, MO, USA), 20 ng/mL basic fibroblast growth factor (PeproTech), B27 (1:50; Invitrogen), and 20 ng/mL epidermal growth factor (PeproTech, Rocky Hill, NJ, USA) were supplemented to DMEM/F12 medium(Invitrogen, Carlsbad, CA, USA). Then we suspended cells infected with LV-shNon and LV-shAspp2 at a concentration of 5000 cells/mL with DMEM/F12 medium. 0.01 μM Simvastatin or DMSO was added to the medium. Five thousand cells were plated onto ultralow attachment plates (Corning, Corning, NY, USA) in different conditions. After one-week of incubation, sphere numbers were counted using an Olympus IX70 microscope.

### Immunofluorescence

Cultured cells were washed 3 times with cold phosphate buffer saline (PBS), fixed with 4% paraformaldehyde, blocked and then incubated with ASPP2 antibody (1:200, A4480, Sigma-Aldrich, antigenic epitope was located at amino acids 691–1128 of ASPP2 of human species) and SREBP-2 antibody (1:150, N-19 sc8151, Santa Cruz, antigenic epitope was located in N-terminus of SREBP-2 of human species) overnight at 4 °C. Corresponding Alexa 555-conjugated and/or Alexa 488-conjugated secondary antibodies (Invitrogen) were used to incubate the cells for 1 h at room temperature, then 4,6-diamidino-2-phenylindole (DAPI; Vector Laboratories Inc., Burlingame, CA) for 5 min. Representative images were captured with LSM710 laser confocal microscope (Fudan University). Immunofluorescence on HCC-LM3 spheres was performed with EpCAM antibody (1:100, #36746, Cell signal technology) using the same method described above, under an Olympus IX70 microscope. Details about Immunofluorescence detection of EpCAM can be found in the supplementary material and method.

### Immunoprecipitation assay

After different treatment, cells were incubated with lysis buffer (50 mM Tris HCl, 150 mM NaCl, 0.1% SDS, 1% NP-40, 0.5% sodium deoxycholate) plus protease inhibitors 1 mM PMSF (Sigma, P7626) and protease inhibitor cocktail tablets (Roche, 04693116001). Cells were lysed for 30 min on ice and centrifuged at 16,000 × *g* for 20 min at 4 °C. The cell lysates were incubated with antibody overnight and the immune complexes precipitated with protein A/G agarose (Santa Cruz, sc-2003) for 3 h at 4 °C. Complexes were washed in lysis buffer (5 × 5 min). Immunoprecipitated proteins were analyzed by Western blotting.

### Immunoprecipitation of nuclear proteins

The nuclear proteins of treated HCC-LM3 or HEK293T cells were isolated by the Nuclear Complex Co-IP Kit (Active Motif, 54001) according to the manufacturer’s instructions. Briefly, the collected cells were resuspended in cold PBS/phosphatase inhibitors, followed by separation of the cytoplasmic fraction and the nuclear fraction, respectively. The separation of nuclear proteins was confirmed by the low expression of cytoplasmic protein α-tubulin (Proteintech, 11224–1-AP) and the high expression of nuclear protein Lamin B1 (Proteintech, 12987–1-AP). The nuclear proteins extract was diluted in lysis buffer, then incubated with antibody overnight and the immune complexes precipitated with protein A/G agarose for 3 h at 4 °C. Immunoprecipitated proteins were analyzed by Western blotting.

### Cell viability and chemo-resistance assays

Cells infected with LV-shAspp2 and LV-shNon were seeded into 96-well plates (5000 cells/well).Then cells were treated with different concentration 5-FU and 10 μM Simvastatin or DMSO for 48 h. Cell viability was measured by MTS assay reagent (CellTiter 96 AQueous One Solution Cell Proliferation Assay; Promega, Madison, WI, USA). The experiments have been repeated at least three times.

### Patient samples

We performed a tissue microarray constructed by Shanghai Weiao Biotechnology Co., Ltd (Weiao Biotechnology Co, Shanghai, China). Eighty primary HCC samples made for microarray were obtained from patients who had undergone curative hepatic resection between 2008 and 2015. The clinicopathologic features of the patients were summarized in Supplementary Table 1. Other details about patient samples can be found in the supplementary material.

### Immunohistochemical staining

The expressions of ASPP2, HMGCR, and HMGCS1 were analyzed with ImageScope system in formalin-fixed, paraffin-embedded sections of primary tumors. Briefly, the slides were dewaxed, hydrated,quenched endogenous peroxidase activity, retrieved antigen, blocked and incubated with the antibody against ASPP2 (1:50, A4480, Sigma-Aldrich), HMGCR (1:50, H300 sc-33827, Santa Cruz), or HMGCS1 (1:50, H-70 sc-33829, Santa Cruz) overnight at 4 °C. Then, sections were rinsed and incubated with the working solution of horseradish peroxidase-labeled goat anti-rabbit for 1 h at 37 °C. After rinse for three times, diaminobenzidine colorimetric reagent solution from Dako (Carpinteria, CA) was used. Subsequently the slides were counterstained by hematoxylin and dehydrated in graded alcohol and mounted. The expression of ASPP2 and HMGCR were scored according to the signal intensity and distribution. Evaluation of immunostaining was independently performed by two experienced pathologists.

### Statistical analysis

We use SPSS16.0 to analyze all data. The *χ*^2^ test was applied to assess qualitative variables 2-tailed Student’s *t* test and Wilcoxon rank sum test were used to valuate quantitative variables. Spearman rank test was used to determine correlations of two variables. Kaplan-Meier analysis was used to achieve Survival analyses of investigated patients. Difference in survival between groups was evaluated by the log-rank test. Univariate and multivariate analyses were based on the Cox proportional hazards regression model. All results were showed as the mean ± SEM. All statistical tests were two-sided, and *P* < 0.05 was considered statistically significant.

Other Material and Methods are available in the supplementary material and Methods.

## Supplementary information

Supplemental Material-revision

declaration of contributions to article
