# Supplementary material for: ASPP2 inhibits tumor growth by repressing the mevalonate pathway in hepatocellular carcinoma
Source: Cell Death Dis. 2019 Nov 4;10(11):830. doi: 10.1038/s41419-019-2054-7 (PMC6828733; doi:10.1038/s41419-019-2054-7)
Supplement: Supplementary file 1 — Supplemental Material-revision [file 41419_2019_2054_MOESM1_ESM.doc]

**Supplementary Material**

**Materials and methods**

**Western blotting.**

Cells were lysed by 1×SDS buffer. SDS–PAGE was used to load and separate proteins, then protein was transferred to PDVF membranes. After probing with primary and secondary antibodies, the antigen-antibody complexes were detected with chemiluminescence reagents (Pierce Biotechnology, Milwaukee, WI, USA). ASPP2 antibody (A4480) used at 1:1000 was purchased from Sigma-Aldrich. HMGCR (H300 sc-33827), HMGCS1 (H70 sc-33829), SREBP-2(N-19, sc8151) antibodies used at 1:1000 were purchased from Santa Cruz. EpCAM (aa1-300, ab223582 ) and EpCAM（aa 250 to the C-terminus, ab71916）antibodies used at 1:100-1:1000 were purchased from abcam. EpCAM（aa 24-93, sc-25308）antibody used at 1:100 was from Santa Cruz.

**Real-time PCR.**

We used the NucleoSpin RNAII (MACHEREY-NAGEL, 740955) to isolate total cellular RNA, and generated first-strand cDNA by PrimeScript RT reagent kit (Takara, DRR037A). The cDNA samples were measured to evaluate mRNA expression by 7500 Fast Real-Time PCR System (Applied Biosystems, Carlsbad, CA, USA) and β-actin was used as an endogenous control. The primer sequences are listed in supplementary table 2. The experiments have been repeated at least three times.

**Lentivirus shRNA and small interfering RNA construction.**

We designed three pairs of cDNA oligonucleotides targeting ASPP2 mRNA expression, using web-based software from Invitrogen (http://rnaidesigner.invitro gen. com/rnaiexpress/) and InvivoGen Inc. (San Diego,CA; http:// www. sirnawizard.com/design. php) . After synthesis, we inserted these double-strand oligos into the vector pENTR/U6 (Invitrogen) and sequenced the resulting plasmids to ensure the shRNA construct targeted human ASPP2 expression or were scrambled,which were generated and designated as LV-shASPP2 and LV-shNon. Then, the plasmids were transfected into HCC-LM3 cells and gene silencing efficiency was validated 48 hours after transfection by Real-time PCR and Western blot The double-strand oligo DNAs with the silencing efficiency for ASPP2 were as follows: #1, 5’-GCTGAGGGAGAAAGAGAAGAA-3’; #2, 5’-CTT TCTTATCTA ATCCTTA-3’; #3, 5’-CAACTAAATTACTGCCTTT-3’. We used scramble shRNA(5’-AATTCTCCGAACGTGTCACGT-3’) as a negative control.

Three pairs of siRNAs targeting SREBP-2 were designed by GenePharma (Shanghai, China). The sequences were used as follows: for SREBP-2-homo-2983 (siSrebp-2), sense 5’ -GCAGAGU UCCUUCUGCCAUTT -3’ and antisense 5’ -AUGGCAG AAGGAACUCUGCTT -3’.

**Lentivirial vector production and Lentivirus infection.**

Lentiviral vectors encoding shRNAs using pLenti6/BLOCKiT-DEST (Invitrogen) were recombined with pENTR-U6-shRNA plasmids by the Gateway cloning system. To produce lentivirus-containing shRNA, we transfected pLenti/BLOCKiT-DEST-sh RNA plasmids and ViraPower Packaging Mix (Invitrogen) into HEK-293T with Lipofectamine 3000 according to the manufacturer’s guidelines. Then, 0.45μm PVDF filters were used to filter the infectious lentivirus after transfection for 48 and 72 h. The recombinant lentiviruses were concentrated 100-fold with complete culture medium and stored at -80 °C. Crystal violet staining was used to evaluate the infectious titer by counting the blue-stained colonies in 293 cells.

**Luciferase reporter plasmids construction and luciferase reporter assays.**

The luciferase reporter plasmids, pGL-hamster-HMGCS1-promoter and pGL-LDLR -promoter were generated as follows. The hamster HMG CoA synthase promoter region (bp -368 to 11) was produced by PCR using hamster genomic DNA as template with AmpliTaq (Promega). The following primers were used: 5’ primer: 5’- GGTACCCACTGACCTTCAATTGGTCAGAGAACC -3’; and 3’ primer: 5’- CTCGAGTGTGCCTCCCGCCCTCAG -3’, where the KpnI site and XhoI were added, respectively. The human LDLR promoter region (bp -308 to -61) was produced by PCR using human genomic DNA as template. The following primers were used: 5’ primer: 5’- GAGCTCACATTTTTAGTGTTTTCCTTTTGAGGCAG AG -3’; and 3’ primer: 5’- CTCGAGCTAGCAGGGGGAGGAGTTTGCAG -3’, where the sacI site and XhoI were added, respectively. As a luciferase reporter plasmid, plasmid pGL-3SRE contained three tandem copies of repeats 2 and 3 of the human LDL receptor promoter, the adenovirus E1b TATA box and 5’ to 3’.The pGL3.0- enhancer (Promega) and the constructed luciferase reporter plasmids were transfected into HCC (3 × 10^4^) cells in 48-well plates with the pRL-TK in triplicate by X-tremeGENE HP DNA Transfection Reagent. Then the cells were collected after 24 h transfection to measure the luciferase activities with the Dual Luciferase Reporter Assay System (Promega).All data are presented as the means ± S.D. The experiments have been repeated at least three times.

**Immunoﬂuorescence**

HCC-LM3 spheres were collected at 1000 rpm for 5 min by centrifugation and wash cell spheres 3 times with 1ml PBS. We collected spheres again and added 500ul 4% paraformaldehyde to fix the spheres for 10 min at 37℃.After that, the spheres were collected and washed with 1ml PBS. We added 500ul of 0.1% Triton X-100 and 2% BSA in PBS to the spheres and incubated the spheres at room temperature for 60 min. The spheres were centrifuged to collect and added EpCAM antibody (1:100, #36746, Cell signal technology) diluted in 500 µL 0.1% BSA for incubating overnight at 4℃. And then, we removed EpCAM antibody and washed the spheres 3 times with 1ml PBS. The spheres were added Alexa 488-conjugated secondary antibody diluted in 500ul 0.1% BSA and incubated for 60 min at room temperature protected from light. Finally, we collected the spheres and stained nuclear with DAPI diluted in 500ul 0.1% BSA for 5 min at room temperature protected from light. Representative images were captured with an Olympus IX70 microscope.

**Tumor xenograft model.**

Male athymic BALB/c nude mice aged 4-6 weeks were purchased from Shanghai Experimental Animal Center of Chinese Academic of Sciences (Shanghai, China). We had four groups,six in each group. Animal were assigned randomly to experimental or control groups by blind selection.All animals were kept and bred in a controlled environment and housed in a pathogen-free barrier facility with a 12h light/dark cycle under a temperature of 21°C and a relative humidity of 50% according to the guidelines of Shanghai Medical Experimental Animal Commission. Animal experiments were approved by Medical Ethics Committee of Shanghai University of Medicine & Health Sciences( 2018-GZR-18-310110196803058627). After infection with LV-shNon and LV-shAspp2 (at a MOI of 50), HCC-LM3 cells (5 × 10^6^) were implanted into the flank of nude mice by subcutaneous injection (male BALB/c nu/nu, six in each group). Tumors were well-established after 7 days. Simvastatin (40 mg/kg body weight) in 0.5% CMC-Na (sodium carboxymethyl cellulose) was given to mice daily by [intraperitoneal injection](../../../../C:/Users/Administrator/AppData/Local/youdao/dict/Application/7.5.2.0/resultui/dict/../dict/result.html%3Fkeyword=intraperitoneal%20injection&lang=en) for 3 weeks. Then the mice were sacrificed and the tumor tissues were isolated for histopathology experiments.

**Plasmids construction and immunoprecipitation Assay**

The plasmid pSREBP-2-HA (2-481 amino acids) which was produced by PCR using human genomic DNA as template with AmpliTaq (Promega). The following primers were used: 5’ primer: 5’- GAATTCTAGACGACAGCGGC GAGC -3’; and 3’ primer: 5’- CTCGAGTTACCGTGAGCGGTCTACC -3’, where the EcoRI site and XhoI were added, respectively. The plasmid pASPP2-V5 was donated by Lu Xin professor (Ludwig Cancer Research Institute, Nuffield Department of Clinical Medicine, University of Oxford).

After different treatment, cells were incubated with lysis buffer (50 mM Tris HCl, 150 mM NaCl, 0.1% SDS, 1% NP-40, 0.5% sodium deoxycholate) plus protease inhibitors 1 mM PMSF (Sigma, P7626) and protease inhibitor cocktail tablets (Roche, 04693116001). Cells were lysed for 30 min on ice and centrifuged at 16000g for 20 min at 4°C. The cell lysates were incubated with antibody overnight and the immune complexes precipitated with protein A/G agarose (Santa Cruz, sc-2003) for 3 hours at 4°C. Complexes were washed in lysis buffer (5×5 min). Immunoprecipitated proteins were analyzed by western blotting.

**Patient samples.**

We performed a tissue microarray constructed by Shanghai Weiao Biotechnology Co., Ltd, China (Weiao Biotechnology Co.,ZL-LVC1605). Weiao Biotechnology Co. was responsible for obtaining informed consent from all subjects. Eighty primary HCC samples made for microarray were obtained from patients who had undergone curative hepatic resection between 2008 and 2015. Patient’s samples were approved to use for research purposes by medical Ethics Committee of Shanghai University of Medicine & Health Sciences( 2018-GZR-18- 310110196803058627). We defined curative resection as complete resection of all tumor nodules and the cut surface being free of cancer by histologic examination. The clinicopathologic features of the patients were summarized in supplementary table 1. Patient follow-up was completed on December 15, 2015. The median follow-up period was 47 months (range, 1–80 months). Overall survival (OS) was defined as the interval between the dates of surgery and death. Recurrence-free survival (RFS) was defined as the interval between the dates of surgery and recurrence, Patients were censored on the date of death or the last follow-up if recurrence was not diagnosed.

**Immunohistochemical staining**.

The expressions of ASPP2, HMGCR and HMGCS1 were analyzed with ImageScope system in formalin-fixed, paraffin-embedded sections of primary tumors. Briefly, the slides were dewaxed, hydrated ,quenched endogenous peroxidase activity, retrieved antigen, blocked and incubated with the antibody against ASPP2 (1:50, A4480,Sigma-Aldrich), HMGCR (1:50,H300 sc-33827,Santa Cruz) or HMGCS1 (1:50,H-70 sc-33829,Santa Cruz)overnight at 4°C. Then, sections were rinsed and incubated with the working solution of horseradish peroxidase-labeled goat anti-rabbit for 1h at 37°C. After rinse for three times, diaminobenzidine colorimetric reagent solution from Dako (Carpinteria, CA) was used. Subsequently the slides were counterstained by hematoxylin and dehydrated in graded alcohol and mounted.

Evaluation of immunostaining was independently performed by two experienced pathologists. The expression of ASPP2 and HMGCR were scored according to the signal intensity and distribution. Briefly, a mean percentage of high tumor cells were determined in at least five areas at ×400 magnification and assigned to one of the five following categories: 0, <5%; 1, 5-25%; 2, 25-50%; 3, 50-75% and 4, >75%. The intensity of immunostaining was scored as follows: 1, weak; 2, moderate and 3, intense. For tumors that showed heterogeneous staining, the predominant pattern was taken into account for scoring. The staining intensity and the percentage of high tumor cells were multiplied to generate a weighted score for each case. Tissues with immunohistochemical scoring≤2 were considered as low, 3-12 as high.

**Microarray analysis**

HCC-LM3 cells were extractd cellular RNA with TRIzol (Thermo Fisher Scientiﬁc) after infected with shASPP2 or shNon lentivirus for 7 days. Then, an RNeasy kit (Qiagen, Hilden, Germany) was used to purify the RNA. NimbleGen Gene Expression Microarray was applied in microarray analysis and scanned by Axon GenePix 4000B microarray scanner. Raw data were measured by NimbleScan software 2.5 .They were deposited in the National Center for Biotechnology Information’s Gene Expression Omnibus (GEO) and are accessible through GEO series accession number: GSE30468. The predicting biologic effects of ASPP2 were evaluated by Gene Ontology (GO) and Kyoto Encyclopedia of Genes and Genomes (KEGG) enrichment analysis of the differentially expressed genes (fold change ⩾ 2, P<0.05) by the ClueGo plugin of Cytoscape (Software version 3.2.3, INSERM UMRS1138, Paris, France). Enrichment analysis was done via functional annotation chart and annotation clustering options, which were limited to GO terms and KEGG pathways in ‘Biologic Process’ categories. P-value< 0.05 was considered signiﬁcant by Fishers exact test. Microarray Expression Analysis ofASPP2 Knockdown in HCC-LM3 cell lines been deposited in the National Center for Biotechnology Information’s Gene Expression Omnibus (GEO) and are accessible through GEO series accession number: GSE30468.

**Supplementary Figures 1**

**
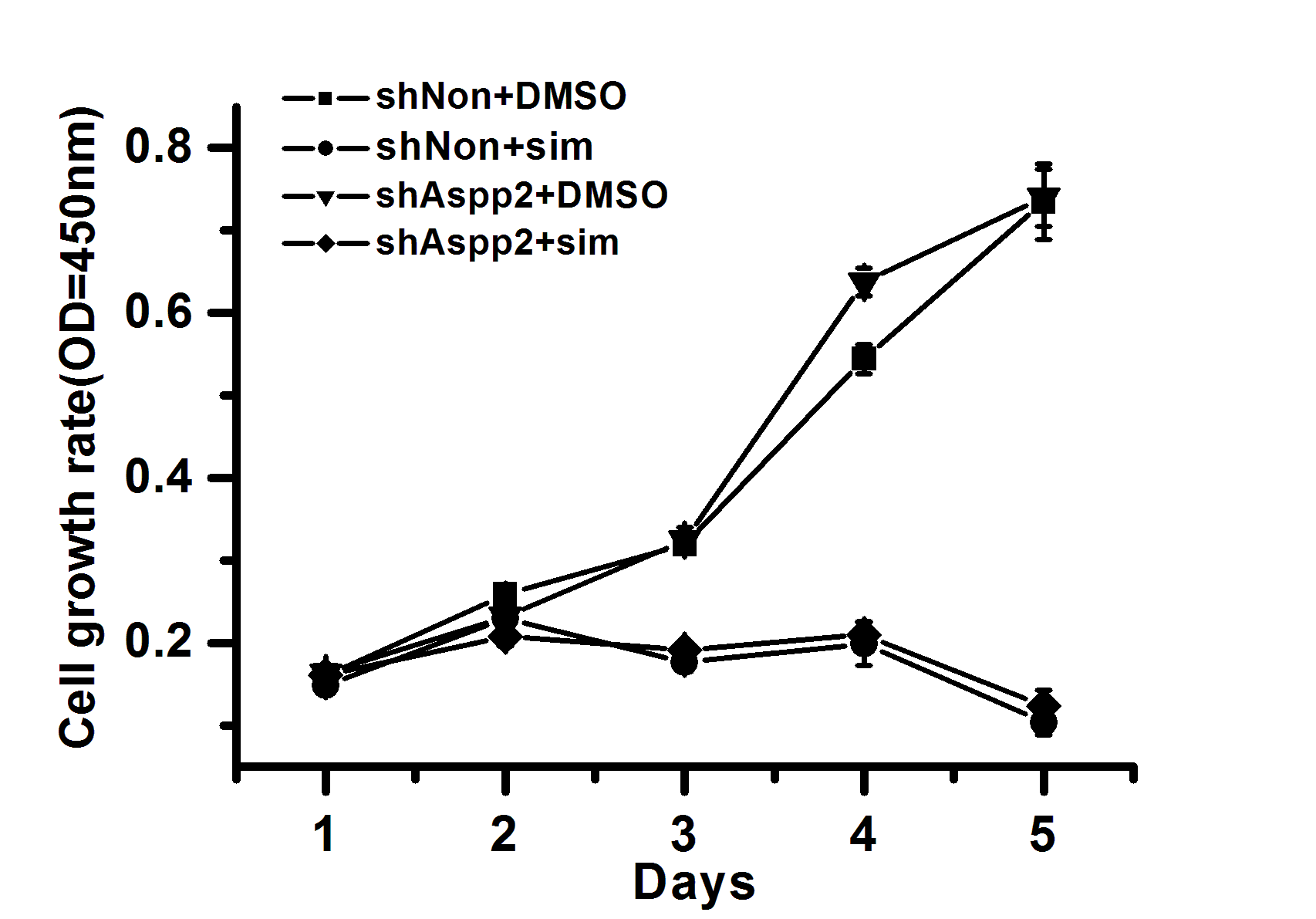
**

**Supplementary Figure 1. ASPP2 has no effect on the proliferation of hepatocarcinoma cells**. HCC-LM3 cells were passaged by lentivirus interference with ASPP2. Then 5×10^3^ cells per well were plated into 96-well plates. Simvastatin (Sim) 10μM and negative control (NC) DMSO solvent were separately added to the medium, and then the cell proliferation rate was measured for 5days at OD 450nm by the CCK-8 reagent.

**Supplementary Figures 2**

**A**

**B**

**
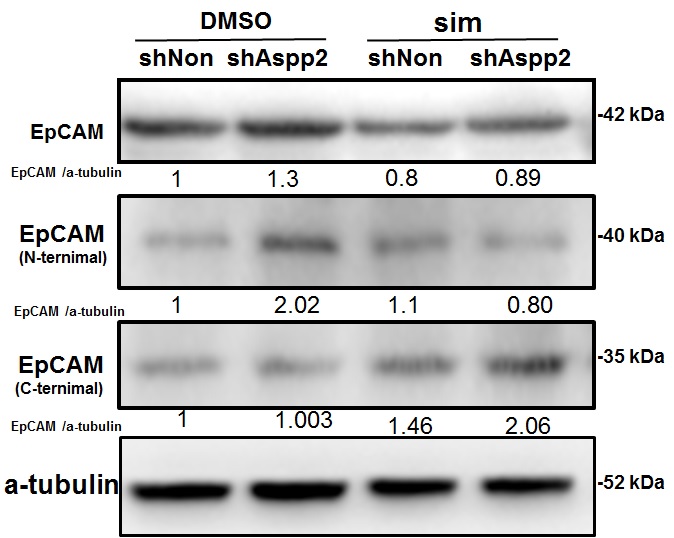
**


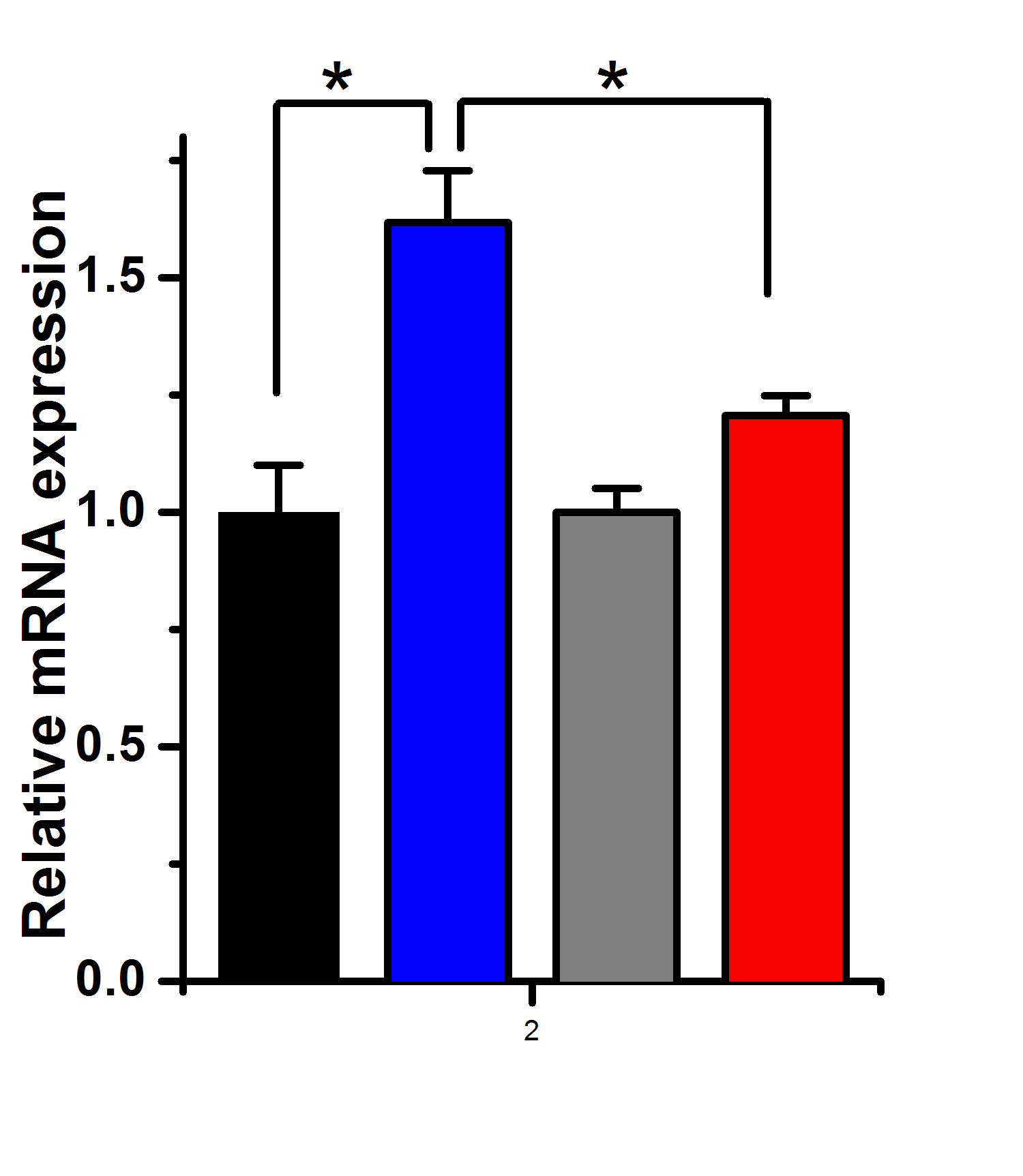


**EpCAM**


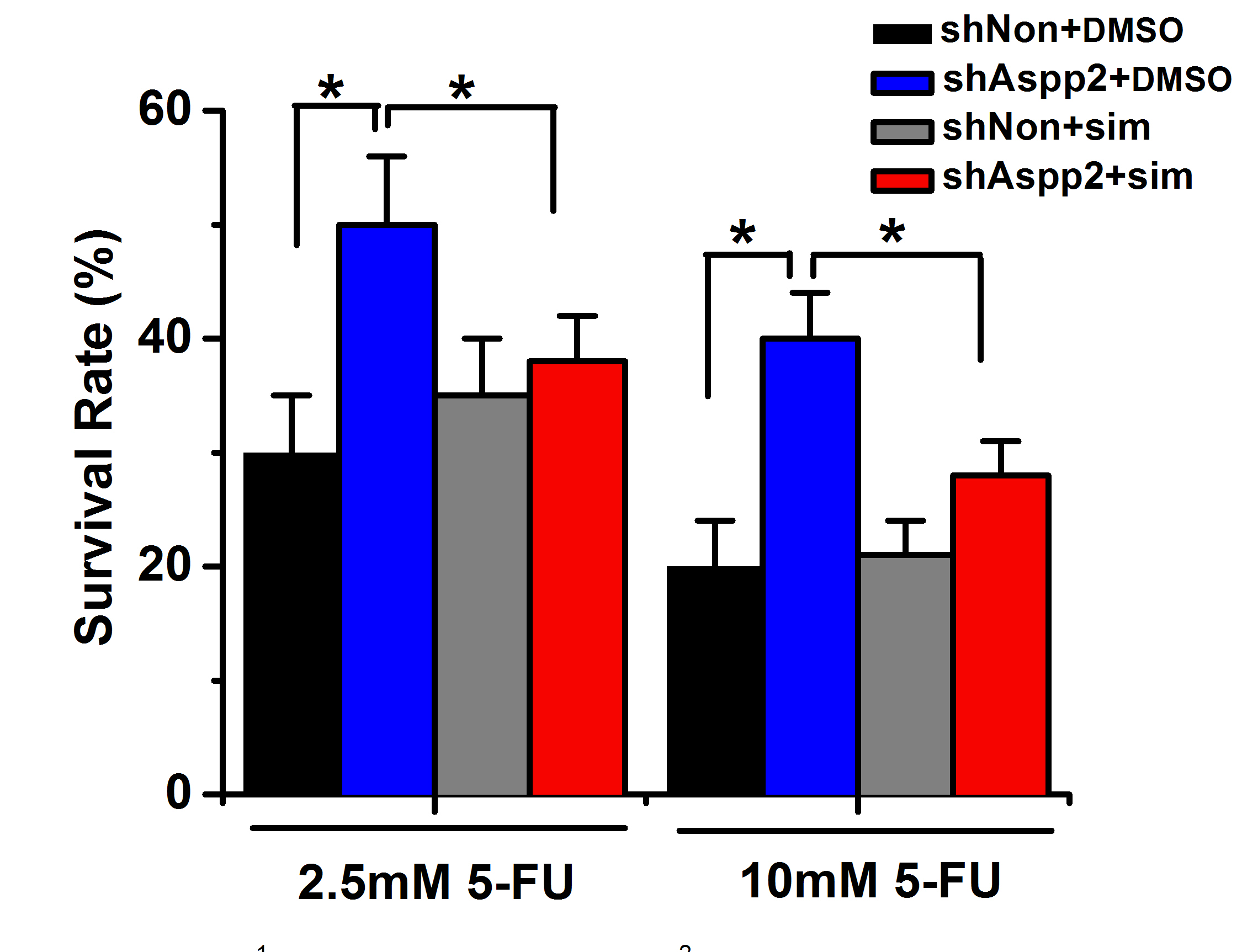


**Supplementary Figure 2. Simvastatin supplementation abrogated ASPP2 depletion-induced EpCAM expression in HCC-LM3 Cell.** **(A)**. qRT-PCR of EpCAM expression in HCC-LM3 infected with lentivirus encoding shRNA and treated with Simvastatin (Sim) 10μM and DMSO solvent. β-actin was used as a control. * indicates *P* <0.05. **(B)**. Corresponding Western-blot of EpCAM with antibodies recognizing whole sequence (aa 1-300), N-terminal (aa 24-93) and C-terminal (aa 250-350) in HCC-LM3.

**Supplementary Figures 3**

**
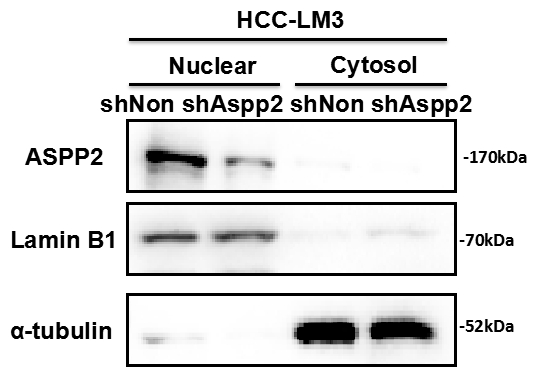
**

**A**

**B**


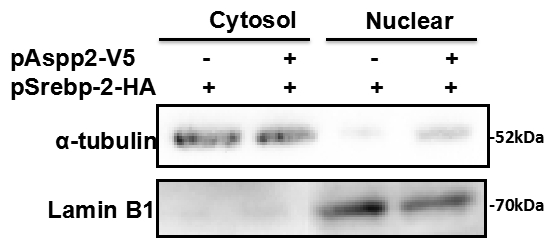

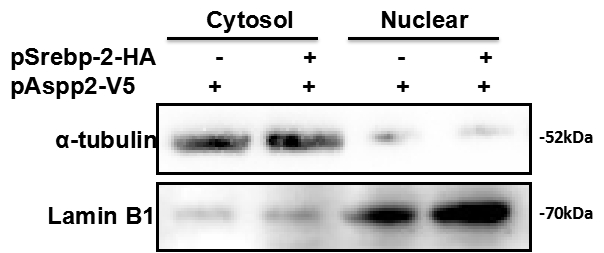
**Supplementary Figures 3. The Identification of nuclear proteins extraction.**

The separation of nuclear proteins was confirmed by the low expression of cytoplasmic protein α-tubulin and the high expression of nuclear protein Lamin B1. **(A).**The expression of cytoplasmic protein and nuclear protein in HCC-LM3cells infected with lentivirus encoding shRNA. **(B-C).** The expression of cytoplasmic protein and nuclear protein in HEK293T cells over-expressioned of Aspp2-V5 plasmid or Srebp-2-HA plasmid.

**Supplementary tables**

**Supplementary Table 1. The clinicopathologic characteristics of 80 cases of HCC**

| **Variables** | **No.of patients(%)** |
| --- | --- |
| **Gender** |  |
| **Male** | **59 (74)** |
| **Female** | **21(26)** |
| **Age (year)** |  |
| ＜50 | **32 (40)** |
| **≥50** | **48(60)** |
| **HBsAg** |  |
| **Positive** | **66 (82)** |
| **Negative** | **14(18)** |
| **AFP (ng/ml)** |  |
| **≤400** | **41 (51)** |
| ＞400 | **39 (49)** |
| **Cirrhosis** |  |
| **－** | **23 (38)** |
| ＋ | **57(62)** |
| **Tumor volume (cm3)** |  |
| ≤5 | **39(49)** |
| >5 | **41 (51)** |
| **AJCC stage** |  |
| Ⅰ-Ⅱ | **34(43)** |
| Ⅲ-Ⅳ | **46(57)** |
| **Recurrence time(months)** |  |
| ≤6 | **28 (35)** |
| >6 | **52(65)** |

Abbreviations: AFP, alpha fetoprotein; HBsAg, hepatitis B surface antigen; AJCC, American Joint Committee on Cancer

**Supplementary Table 2. Primers used in this study**

| **Primers for Real-time PCR** | | |
| --- | --- | --- |
| **Protein** | | **Sequence (5’→3’)** |
| ASPP2  HMGCR | F  R  F | GAAGACTCGGTGAGCATGCG  GCGATACGCTCTGAGCCAGT  TGATTGACCTTTCCAGAGCAAG |
|  | R | CTAAAATTGCCATTCCACGAGC |
| HMGCS1 | F | GATGTGGGAATTGTTGCCCTT |
|  | R | ATTGTCTCTGTTCCAACTTCCAG |
| MVK | F | GGAGCAAGGTGATGTCACAAC |
|  | R | CGGCAGATGGACAGGTATAAGT |
| MVD | F | GGACCGGATTTGGCTGAATG |
| FDFT1  IDI1-F  SREBP-1  SREBP-2  EpCAM | R  F  R  F  R  F  R  F  R  F  R | CCCATCCCGTGAGTTCCTC  CCACCCCGAAGAGTTCTACAA  TGCGACTGGTCTGATTGAGATA  TCCATTAAGCAATCCAGCCGA  CCCAGATACCATCAGACTGAGC  ACAGTGACTTCCCTGGCCTAT  GCATGGACGGGTACATCTTCAA  AACGGTCATTCACCCAGGTC  GGCTGAAGAATAGGAGTTGCC  AATCGTCAATGCCAGTGTACTT  TCTCATCGCAGTCAGGATCATAA |
| β-actin | F | CGTGGACATCCGTAAAGACC |
|  | R | ACATCTGCTGGAAGGTGGAC |

**Supplementary Table3**. Antibodies used in this study

| **Protein** | **Usage** | **Antibody Dilution** |
| --- | --- | --- |
| ASPP2 | WB | A4480, Sigma-Aldrich 1:1000 |
| ASPP2 | IP | A4480, Sigma-Aldrich 1:40 |
| ASPP2 | IF | A4480, Sigma-Aldrich 1:200 |
| ASPP2 | IHC | A4480, Sigma-Aldrich 1:50 |
| HMGCR | WB | H300 sc-33827, Santa Cruz 1:1000 |
| HMGCR | IHC | H300 sc-33827, Santa Cruz 1:40 |
| HMGCS1 | WB | H70 sc-33829,Santa Cruz 1:1000 |
| HMGCS1 | IHC | H70 sc-33829, Santa Cruz 1:40 |
| SREBP-2 | WB | N-19 sc-8151, Santa Cruz 1:1000 |
| SREBP-2 | IF | N-19 sc-8151, Santa Cruz 1:200 |
| EpCAM | IF | 36764,Cell signal technology 1:100 |
| EpCAM（aa 1-300）  EpCAM（C-teminal）  EpCAM（N-teminal） | WB  WB  WB | ab223582,abcam 1:1000  ab71916 ,abcam 1:1000  sc-25308, Santa Cruz 1:100 |
| GAPDH | WB | KC5G5,KangChen Bio-tech 1:2000 |
| HA | WB | H9658, Sigma-Aldrich 1:1000 |
| HA | IP | H9658, Sigma-Aldrich 1:40 |
| V5 | WB | V8137, Sigma-Aldrich 1:1000 |
| V5 | IP | V8137, Sigma-Aldrich 1:40 |
| Lamin B1 | WB | 12987-1-AP,Proteintech 1:1000 |
| a-tubulin | WB | 11224-1-AP,Proteintech 1:2000 |

**Note:**

Secondary antibodies used in Western blot analysis, goat anti-rabbit IgG-HRP (sc-2030), goat anti-mouse IgG-HRP(sc-2031) and donkey anti-goat IgG-HRP (sc-2020) were purchased from Santa Cruz Biotechnology. Secondary antibodies used in immunofluorescence, Alexa Fluor 555 donkey anti-goat IgG, Alexa Fluor 488 donkey anti-mouse IgG were purchased from Life technologies. Abbreviations: WB, Western blotting; IF, immunofluorescence; IHC, immunohistochemistry; IP, immunoprecipitation.
