# Supplementary material for: ASPP2 inhibits tumor growth by repressing the mevalonate pathway in hepatocellular carcinoma
Source: Cell Death Dis. 2019 Nov 4;10(11):830. doi: 10.1038/s41419-019-2054-7 (PMC6828733; doi:10.1038/s41419-019-2054-7)
Supplement: Supplementary file 2 — declaration of contributions to article [file 41419_2019_2054_MOESM2_ESM.pdf]

DECLARATION OF CONTRIBUTIONS TO ARTICLE

ADMC

Manuscript Number:	Journal Name:	
CDDIS-19-1858R	*Cell Death & Differentiation*	(the 'Journal')

Proposed Title of the Contribution:

ASPP2 inhibits tumor growth by repressing the mevalonate pathway in hepatocellular carcinoma (the 'Contribution')

Author(s):

Beibei Liang, Rui Chen, Shaohua Song, Hao Wang, Guowei Sun, Hao Yang, Wei Jing, Xuyu Zhou,Zhiren Fu,Gang Huang, Jian Zhao (the 'Authors')

For all *CDD* articles, each person named as an author in the published version must be able to show he or she has contributed substantially to the article.

Authorship credit should be based on 1) substantial contributions to conception and design, acquisition of data, or analysis and interpretation of data; 2) drafting the article or revising it critically for important intellectual content; and 3) final approval of the version to be published. Authors should meet conditions 1, 2 and 3.

Any person who cannot be shown to have made a substantial contribution to the article cannot be listed as an author in the final version. The name of any person who is deemed to have made a minor contribution can, however, appear in the Acknowledgments section of the article.

Please complete the table below to indicate the contributions of all named authors to the manuscript.

Author Full Name:	Specification of Contribution to the Manuscript:
Beibei Liang	acquisition of data (gene expression and clinical data analysis, animal study), drafting the manuscript
Rui Chen	acquisition of data (regulation pathway and gene expression analysis)
Shaohua Song	acquisition of data (animal study and clinical data analysis)
Hao Wang	acquisition of data (regulation pathway and protein interaction analysis)
Guowei Sun	acquisition of data (animal study)
Hao Yang	acquisition of data (protein interaction analysis)
Wei Jing	acquisition of data (clinical data analysis)
Xuyu Zhou	acquisition of data (clinical data support) and revising the paper for its clinical importance
Zhiren Fu	acquisition of data (clinical data support) and revising the paper for its clinical importance
Gang Huang	study design, data analysis, financial and technical support, and final approval for publication
Jian Zhao	study design, drafting the article, data analysis, financial and technical support, and final approval for publication

Please complete the table below to indicate the contributions of all named authors to the figures.

Figure 1:

Figure 1A and B: Rui Chen and Beibei Liang performed gene expression analysis .
Figure 1C-E: Beibei Liang and Hao Wang performed RT-PCR and Western Blotting.
Jian Zhao analysed and interpreted the data.

Figure 2:

Figure 2A and C: Rui Chen and Hao Wang performed sphere assay and immuno-staining analysis.
Figure 2B and D: Beibei Liang and Rui Chen performed RT-PCR and cell survival assay.
Jian Zhao analysed and interpreted the data.

Figure 3:

Figure 3A-C: Beibei Liang, Shaohua Song and Guowei Sun conducted animal study.
Jian Zhao and Gang Huang analysed and interpreted the data.

Figure 4:

Figure 4A: Rui Chen and Hao Wang performed confocal analysis.
Figure 4B-D: Hao Yang and Hao Wang performed Co-IP analysis.
Figure 4E: Rui Chen and Hao Wang performed promoter activity analysis.
Figure 4F-G: Beibei Liang performed sphere assay and Western Blotting.
Jian Zhao and Gang Huang analysed and interpreted the data.

Figure 5:

Figure5: Beibei Liang, Shaohua Song and Wei Jing performed IHC and survival assay on clinical samples.
Xuyu Zhou and Zhiren Fu provided support for clinical data collection and critical revision for clinical analysis.
Jian Zhao and Gang Huang analysed and interpreted the data.

Figure 6:

Table 1-3: Beibei Liang, Shaohua Song and Wei Jing performed clinical data assay.
Xuyu Zhou and Zhiren Fu provided support for clinical data collection and critical revision for clinical analysis.
Jian Zhao and Gang Huang analyzed and interpreted the data.

Signed for and on behalf of the Author(s):	Print Name:	Date:
Jian Zhao	Jian Zhao	19-09-2019
